# Reemergence of Scabies Driven by Adolescents and Young Adults, Germany, 2009–2018

**DOI:** 10.3201/eid2706.203681

**Published:** 2021-06

**Authors:** Felix Reichert, Maike Schulz, Elke Mertens, Raskit Lachmann, Anton Aebischer

**Affiliations:** European Centre for Disease Prevention and Control, Stockholm, Sweden (F. Reichert);; Robert Koch Institut, Berlin, Germany (F. Reichert, R. Lachmann, A. Aebischer);; Central Research Institute of Ambulatory Health Care in Germany, Berlin, Germany (M. Schulz);; Public Health Agency of Lower Saxony, Hanover, Germany (E. Mertens)

**Keywords:** scabies, scabicides, incidence, Germany, Europe, young adults, treatment failure, parasites

## Abstract

To validate anecdotal evidence on scabies infestations, we analyzed inpatient and outpatient claims data in Germany. Scabies diagnoses increased 9-fold and treatment failure 4-fold during 2009–2018, driven mainly by persons 15–24 years of age. Prevention and control in young adults appear key because of these persons’ high mobility and social connectivity.

Anecdotal evidence from clinicians in Germany suggests an increase in scabies; sales of scabicides by pharmacies in Germany have quadrupled during 2012–2017 (*1**,**2*). In addition, clinicians and scientists have raised concerns about resistance to standard treatment (*3*). In Germany, scabies is not reportable, and no recent national incidence estimates exist. 

Scabies is diagnosed clinically, but confirmation through skin scrapings or dermatoscopy is not always performed in Germany (*1*). The national guideline recommends a single application of permethrin 5% cream for common scabies (*4*). Ivermectin, licensed in Germany in 2016, is recommended in cases of crusted scabies, immunosuppression, and contraindications for topical treatment (*4*). A second application is recommended after 7–15 days in outbreaks and patients with crusted scabies, immunosuppression, or persistent infestation. We investigated incidence of scabies in Germany for 2009–2018.

## The Study

We analyzed claims data of outpatients insured by German statutory health insurance (SHI) funds, which applies to ≈90% of the population of Germany (*5*). Information on all ambulatory consultations and filled prescriptions of SHI-covered patients are gathered and stored up to 10 years for the SHI Physicians’ Association by the Central Research Institute of Ambulatory Health Care. 

We defined a case as any patient consultation during 2009–2018 marked with code B86, “scabies,” from the International Classification of Diseases (ICD), 10th Revision. We counted patients with repeat consultations only once per year. We excluded cases with missing or implausible age or sex information. We extracted, aggregated, and analyzed time of diagnosis, age, sex, and area of residence. We calculated incidence as number of cases per 100,000 SHI members per year. We also analyzed prescribing data for allethrin, benzyl benzoate, crotamiton, ivermectin, lindane, and permethrin linked to cases. We assumed treatment failure and defined repeated prescriptions if a patient received prescriptions for 2 scabicides within a year >28 days apart, regardless of substance (*6*). Use of claims data is regulated by the Code of Social Law (Sozialgesetzbuch) in Germany; ethics approval and informed consent are not required. 

**Figure 1:**
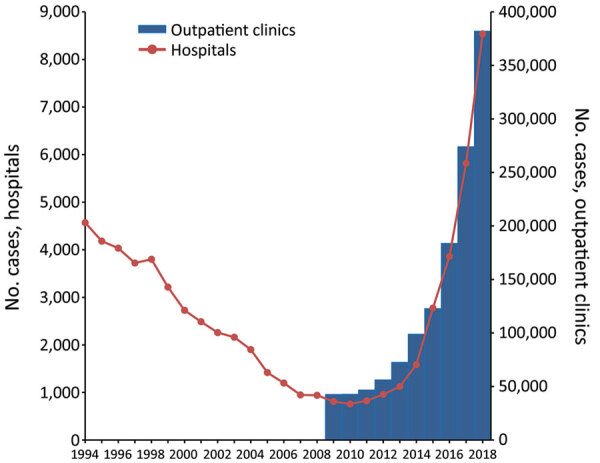
Scabies diagnoses in outpatient clinics among members of statutory health insurance funds and in hospitals, by year, Germany, 1994–2018. Scales for the *y*-axes differ substantially to underscore patterns but do not permit direct comparisons.

In 2009, German SHI funds had 70,011,508 members, and scabies was diagnosed 42,585 times in physician practices, out-of-hours services, and hospital emergency departments in the ambulatory setting. In 2018, diagnoses were 382,043 for 72,802,098 members, a 9-fold increase in 9 years (Figure 1) and an overall incidence of 525/100,000 persons. 

**Figure 2:**
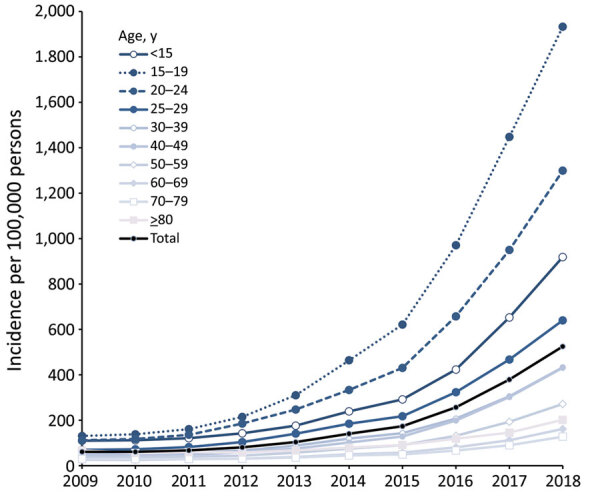
Incidence of scabies diagnoses in outpatient clinics per 100,000 members of statutory health insurance funds, by age group, Germany, 2009–2018.

The highest incidence and a >11-fold increase during 2009–2018 were observed in persons 15–19 and 20–24 years of age (Figure 2). The increase in incidence was more pronounced in boys and men, especially for those 15–19 (23% lower incidence than girls and women in 2009 vs. 7% higher in 2018) and 20–24 years of age (5% lower incidence in 2009 vs. 35% higher in 2018). Incidence showed regional differences in 2018; incidence was higher in northern and western federal states (Bremen, Hamburg, North Rhine-Westphalia, and Schleswig-Holstein) than in the other states (Appendix Figure). Reasons for these differences are unknown.

Filled prescriptions for scabicides increased 14-fold during 2009–2018, from 57,482 to 815,952. Our review of preliminary data through mid-2020 suggests a continued increase. Permethrin was the most commonly used scabicide (90% of prescriptions in 2009; 71% in 2018). Since its licensing in 2016, ivermectin has become the second most prescribed scabicide (27% of prescriptions in 2018). Repeated prescriptions became more common over time (6% of all patients in 2009 vs. 23% in 2018); in 2018, the highest proportion of these was seen in patients 15–19 years of age (Table).

**Table:** Number of outpatients with repeated scabies treatment prescriptions after >28 d, by age group, Germany, 2009 and 2018

Age group, y	No. patients with scabicide prescription				No. patients with repeated scabicide prescription				Percentage of patients with repeated prescriptions	
	2009		2018		2009		2018		2009	2018
<5	3,844		29,255		248		6,999		6	24
5–9	4,687		33,002		264		7,988		6	24
10–14	3,781		37,693		230		10,298		6	27
15–19	4,900		71,610		314		21,100		6	29
20–24	4,350		57,271		225		14,351		5	25
25–29	3,017		33,830		131		7,072		4	21
30–34	2,235		24,466		106		4,630		5	19
35–39	2,215		23,871		92		4,565		4	19
40–44	3,091		23,327		131		4,689		4	20
45–49	3,062		25,006		133		5,108		4	20
50–54	2,354		24,197		125		4,405		5	18
55–59	1,821		17,485		123		2,941		7	17
60–64	1,274		11,534		87		1,917		7	17
65–69	1,376		8,043		110		1,376		8	17
70–74	1,509		5,778		109		906		7	16
75–79	1,230	6,920			102	1,105			8	16
≥80	3,321	15,998			230	2,228			7	14
Total	48,067	449,286			2,760	101,678			6	23

To extend the time period covered, we included diagnostic data from hospitals in Germany available starting in 1994 (most acute care hospitals; n = 1,942 in 2017) (*7*). We extracted the number of hospitalized patients with scabies by age group from publicly available databases (*7*) on the basis of code 133, “acariasis” (1994–1999), from ICD, 9th Revision, and code B86, “scabies” (2000–2018), from ICD, 10th Revision.

As with outpatients, scabies was increasingly diagnosed in inpatients beginning in 2010, indicating the value of these data as a proxy for the time period 1994–2009 (Figure 1). During the period 1994–2018, case numbers decreased for the first 16 years, but the proportion of cases in persons 15–24 years of age persistently increased, from 9% to 20% (Figure 1). The proportion of cases in persons <15 years of age decreased at the same time, from 59% to 46%.

## Conclusions

This study, which analyzed claims data, shows a reemergence of scabies in Germany, with a shift in age distribution and an increase in treatment failure. After a decrease in scabies cases from the mid-1990s through 2008, scabies diagnoses increased 9-fold and scabicide prescriptions 14-fold during 2009–2018. 

Two main observations may help explain this increase. First, persons 15–24 years of age have been particularly affected by the increase in recent years. In contrast, globally, prevalence is highest in young children, as it was in Germany in the mid-1990s (*8*). A recent report from Norway highlights that this age shift is observed internationally (*9*). Increased incidence was reported from Croatia (*10*), and diagnostic data in the Netherlands since 2012 have shown an incidence increase, including outbreaks not only in care facilities but also among students (E. Fanoy, Public Health Service, Rotterdam-Rijnmond, the Netherlands, pers. comm., May 15, 2020). Adolescents and young adults, particularly male, show the highest social connectivity (*11*). For this generation of young adults, mobility is not limited by country borders, which complicates contact tracing (*12*). The possible contribution of cross-border transmission to the international reemergence of scabies must be investigated in further studies. Moreover, young adults have the highest proportion of new sex partners within the previous year (*13*). Scabies can be seen as a sexually transmitted infection, but unlike other such infections, scabies cannot be prevented by condom use. However, intimate contacts that favor scabies transmission are not limited to sexual intercourse and may be related to other behaviors linked to social connectivity and more common in this age group, such as shared housing and couch surfing.

Second, repeated scabicide prescriptions are especially frequent in adolescents and young adults. Failure of first-line treatments is observed in 6%–8% of all patients in clinical trials (*14*). Although studies are needed for direct information on treatment failure, repeated prescriptions (as a proxy for treatment failure) occurred in comparable proportion in Germany in 2009 (Table). Alarmingly, since 2009 this proportion has more than doubled in all age groups but has more than quadrupled in adolescents and young adults. Although we cannot exclude reduced drug efficacy, we consider low therapeutic compliance and higher risk of reinfestation resulting from inefficient treatment of contact persons in this group to be the more relevant cause. Young adults show low medication adherence and high social connectivity, in particular within their own peer group (*11**,**15*).

Our analyses of claims data have limitations, including unknown extent of misdiagnosis and incorrect coding, lack of information on patients’ compliance and medical history, and retreatment because of persisting symptoms rather than persisting infestation. In addition, recently raised awareness may have led to more diagnosed cases. Furthermore, prescriptions that were not reimbursed by the statutory health insurance but were purchased by the patients themselves in the pharmacy were not included in the database.

In summary, epidemiology of scabies in Germany has changed, and persons 15–24 years of age are the new most-affected age group. This group likely drives a current epidemic with uncurbed dynamics. Treatment failure is particularly high in this age group. Improvements in disease management strategies to address and improve awareness, compliance, risk behavior, and contact identification in peer groups are urgently needed. 

Appendix Additional information on reemergence of scabies driven by adolescents and young adults, Germany, 2009–2018.
