# Supplementary material for: Reemergence of Scabies Driven by Adolescents and Young Adults, Germany, 2009–2018
Source: Emerg Infect Dis. 2021 Jun;27(6):1693–6. doi: 10.3201/eid2706.203681 (PMC8153882; doi:10.3201/eid2706.203681)
Supplement: Appendix — Additional information on reemergence of scabies driven by adolescents and young adults, Germany, 2009–2018. [file 20-3681-Techapp-s1.pdf]

Article DOI: https://doi.org/10.3201/eid2706.203681

Reemergence of Scabies Driven by Adolescents and Young Adults, Germany, 2009–2018

Appendix

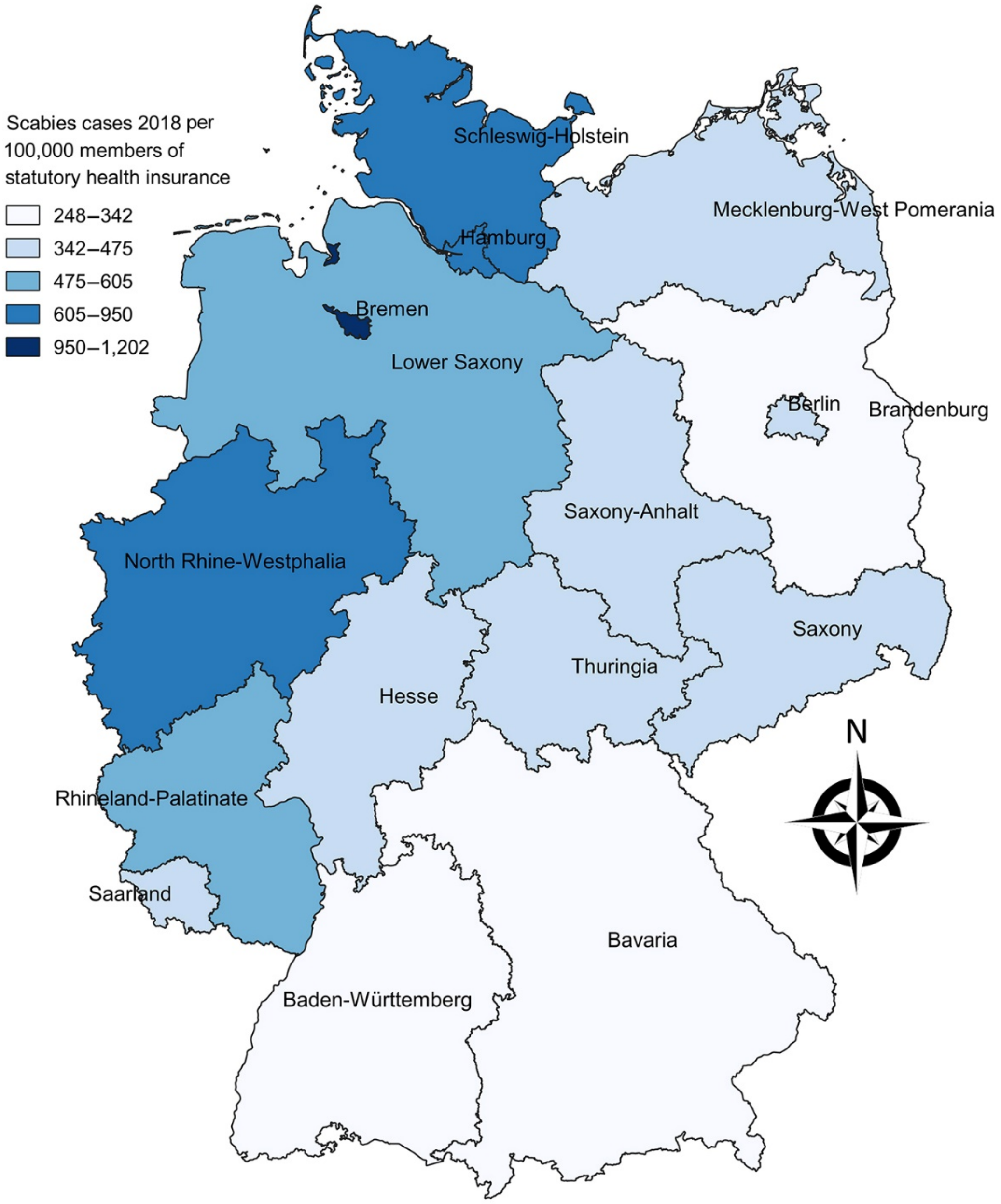

Appendix Figure. Incidence of scabies diagnoses in outpatient clinics per 100,000 members of statutory health insurance funds, by federal state, Germany, 2018.
